# Ultrasonographic Measurement of Anterior Fontanelle Size in Infants with Deformational Plagiocephaly

**DOI:** 10.3390/jcm13175012

**Published:** 2024-08-24

**Authors:** Jae Hee Lee, Gi-Young Park, Dong Rak Kwon

**Affiliations:** Department of Rehabilitation Medicine, School of Medicine, Daegu Catholic University, Nam-gu, Daegu 42472, Republic of Korea; wowchon@dcmc.co.kr (J.H.L.); parkgy@cu.ac.kr (G.-Y.P.)

**Keywords:** deformational plagiocephaly, anterior fontanelle size, cranial vault asymmetry, developmental delay, ultrasonography

## Abstract

**Background/Objectives**: We aimed to investigate the relationship between deformational plagiocephaly (DP) severity and anterior fontanelle size and to explore the connection between fontanelle size and developmental delay. **Methods**: We enrolled 189 (122 boys and 67 girls; mean corrected age, 119.79 days) of the 256 infants who visited our clinic for plagiocephaly between March 2022 and June 2023. This study analyzed the correlation between cranial vault asymmetry (CVA) and anterior fontanelle size as measured using skull anteroposterior (AP) radiography and ultrasonography. The severity of DP was graded from minimal to severe based on the Argenta classification. Infants were grouped according to CVA severity as follows: Group 1 (CVA ≤ 5 mm), Group 2 (5 mm < CVA < 10 mm), and Group 3 (CVA ≥ 10 mm). Additionally, 40 infants underwent the Denver Developmental Screening Test II (DDST-II) for neurodevelopmental delays and were divided into groups based on the presence or absence of developmental delays for fontanelle size comparison. **Results**: Age showed a significant negative correlation with fontanelle size (correlation coefficient −0.234, *p* < 0.05), indicating that fontanelle size decreases as infants age. No significant differences in fontanelle size were observed among the three CVA groups (*p* = 0.074) or between the developmentally delayed and non-delayed groups (*p* = 0.09). This study found no correlation between CVA and fontanelle size or between fontanelle size and developmental delay. **Conclusions**: The findings show that, while anterior fontanelle size decreased with age, there was no significant correlation between the fontanelle size and the severity of deformational plagiocephaly or developmental delays.

## 1. Introduction

Plagiocephaly, characterized by an asymmetrical skull shape with flattening, includes two primary types: synostotic and non-synostotic plagiocephaly, also referred to as deformational or positional plagiocephaly [1]. Deformational plagiocephaly (DP) typically manifests in infants aged > 6 weeks; it is characterized by an altered skull shape yet with normal and open cranial sutures, indicating a lack of craniosynostosis [2]. Previously viewed mainly as a cosmetic concern, recent research has indicated that infants with DP may also face various developmental challenges that may require intervention during their primary school years [3,4,5]. The prevalence of DP varies widely, ranging from 5% to 48% [2]. Several factors contribute to this condition, including supine sleeping positions and intrauterine constraints, such as those occurring in multiple births, prematurity, and obstetric factors, such as assisted vaginal delivery using forceps or vacuum, as well as torticollis [2,6,7,8,9,10].

The diagnosis of DP is based on clinical and physical examinations, with tools such as diagonal calipers and three-dimensional scanners commonly used to measure the diagonal lengths of the head and to determine the severity of plagiocephaly [11,12]. Radiographic imaging also aids in the diagnosis by employing modalities such as plain radiography, ultrasonography, computed tomography (CT), and magnetic resonance imaging (MRI) to detect craniosynostosis [13,14,15,16]. Ultrasonography is particularly notable because of its ease of use, non-invasiveness, and minimal radiation exposure. It is used in various clinical situations, including the evaluation of musculoskeletal issues, fetal examinations, and gastrointestinal system assessments. The use of CT to evaluate synostotic plagiocephaly in infants is burdensome owing to harmful radiation [15,17,18]. Previous studies have demonstrated that ultrasonography, with its high sensitivity and specificity for diagnosing synostosis, can serve as an effective screening tool prior to conducting a three-dimensional CT, which remains the gold standard for diagnosing synostotic plagiocephaly [10,14,15,16,19,20].

The anterior fontanelle is the largest among the fontanelles and is located between the frontal and parietal bones; it is associated with several medical conditions, including delayed brain growth, craniosynostosis, thyroid disorders, elevated intracranial pressure, and genetic diseases [21]. Various studies have reported a wide range of anterior fontanelle sizes at birth, from 0.6 cm to 3.6 cm [22] and from 2.31 cm to 2.85 cm [23]. The closure time of the anterior fontanelle also varies, with early closure sometimes considered a physiological phenomenon [21]. However, previous studies have indicated that premature closure of the anterior fontanelle can impede development in certain areas, such as motor skills, personal–social interactions, and cognitive function [24,25]. However, studies focusing on the relationship between the anterior fontanelle and developmental delay, particularly in patients with DP, are limited.

A recent study analyzed the effect of helmet therapy on DP relative to the size of the anterior fontanelle [26]. A larger anterior fontanelle size was found to predict more effective helmet therapy outcomes. Despite these insights, there is a notable absence of standardized data on anterior fontanelle size for each age group among patients with DP, thereby underscoring the need for further research. From this perspective, our study measured and analyzed anterior fontanelle size across different age groups in infants with DP. We also analyzed the relationship between the severity of DP and anterior fontanelle size through the use of ultrasonography, which is also employed in the assessment of cranial sutures. To the best of our knowledge, no previous study has used ultrasonography to simultaneously assess both anterior fontanelle size and synostosis in patients with DP. Therefore, this study aimed to explore the correlation between the severity of DP and anterior fontanelle size and to further investigate the potential relationship between anterior fontanelle size and developmental delay.

## 2. Materials and Methods

We enrolled 189 (122 boys and 67 girls; average corrected age, 119.79 days) of the 256 infants who visited our outpatient clinic for plagiocephalopathy between March 2022 and June 2023. All infants underwent a pretreatment evaluation during their first outpatient visit. The infants met the following criteria: exhibiting cranial asymmetry characterized by flattening on one side of the skull and capable of undergoing caliper cephalometry and ultrasonographic measurements. Infants diagnosed with craniosynostosis using ultrasonography were excluded.

**Figure 1:**
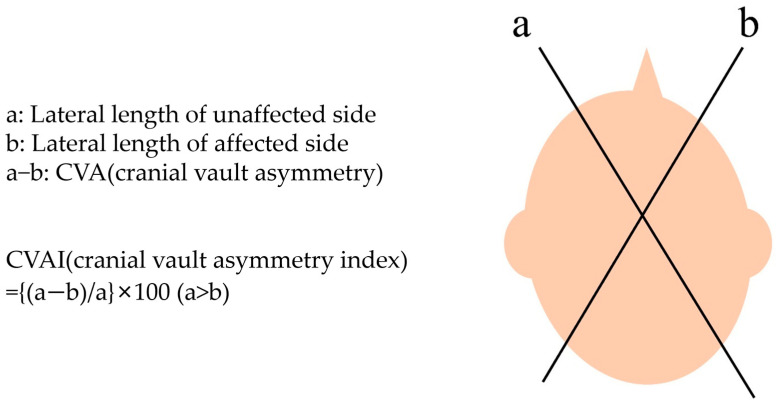
Calculation of the cranial vault asymmetry index using caliper cephalometry. This method involved measuring the lateral length, defined as the distance between the frontozygomatic and contralateral occipital bones, on both the unaffected and affected sides of the skull. Cranial vault asymmetry (CVA) was calculated by subtracting the lateral length on the affected side (b) from the unaffected side (a). Subsequently, the cranial vault asymmetry index (CVAI) was computed by dividing the resulting CVA (a−b) by the lateral length of the unaffected side (a) and then multiplying by 100 to express it as a percentage.

Head length and circumference were measured via caliper cephalometry by a pediatric physiatrist [27,28]. The lateral length, defined as the distance between the frontozygomatic and contralateral occipital bones, was measured on both the unaffected and affected sides of the skull. Cranial vault asymmetry (CVA) was determined by subtracting the lateral length of the affected side (b) from that of the unaffected side (a). The cranial vault asymmetry index (CVAI) was calculated by dividing CVA (a, b) by the lateral length of the unaffected side (a) and multiplying by 100 (Figure 1) [14].

**Figure 2:**
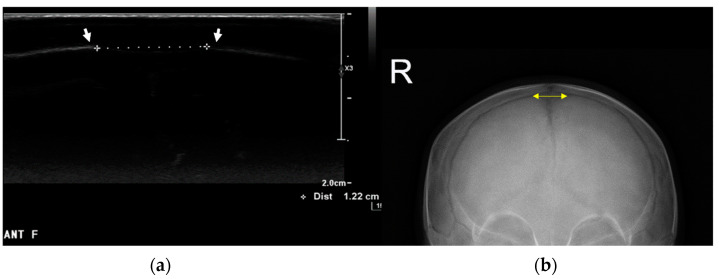
Measurement of anterior fontanelle size using ultrasonography (**a**) and an anterior–posterior (AP) radiograph of the skull (**b**). (**a**) The size of the anterior fontanelle was defined as the longest transverse distance between the skull bones, as indicated by the white arrows in the ultrasound image; (**b**) similarly, in the skull AP radiograph, the size was determined by the longest transverse distance between the ends of each coronal suture, marked by the yellow arrows.

Ultrasonographic evaluations and anteroposterior (AP) radiographs of the skull were obtained by a physiatrist with 20 years of experience in musculoskeletal ultrasonographic procedures to rule out craniosynostosis and to measure anterior fontanelle size. An EPIQ 5 ultrasonographic system (Philips Healthcare, Andover, MA, USA) equipped with a 5–18 MHz multifrequency linear transducer was used. During ultrasonographic evaluation, the entire skull, spanning from the mastoid to the posterior fontanelle, was scanned in all children. Transverse scanning of both lambdoid sutures was performed, and a short cine loop of the flattest portions of the occipital bone was recorded. We also analyzed the correlation between the CVA and anterior fontanelle size. Anterior fontanelle size was defined as the longest transverse distance between the skull bones at the frontal head on ultrasonography and as the longest transverse length on AP radiographs of the skull, according to a previous study (Figure 2) [26]. Ultrasonographic assessments were performed by the same researcher (KDR); two measurements were obtained to verify the intra-rater reliability.

The severity of DP was evaluated using a grading system consisting of five grades progressing from minimal to severe deformation, as reported previously [29]. The infants were divided into three groups based on the degree of CVA. Anterior fontanelle size was compared among the three groups as follows: Group 1 included 43 infants (CVA ≤ 5 mm); Group 2 included 105 infants (5 mm < CVA < 10 mm); and Group 3 consisted of 41 infants (CVA ≥ 10 mm).

Of the 189 infants, 40 underwent the Denver Development Screening Test II (DDST-II) for neurodevelopmental delays. The DDST-II is a widely used tool for screening developmental delays and is employed in most hospitals in Korea [30,31,32,33]. The test evaluated four developmental domains: personal–social, fine motor–adaptive, language, and gross motor. Each test item was assessed as “pass”, “fail”, or “refusal”. A delay is defined as a child failing a test item that 90% of their age-mates pass, whereas caution is defined as failing a test item that falls between 75% and 90% of their age-mates. We categorized a child’s test performance as follows: “normal” indicates no delay in any domain and no more than one caution; “questionable” indicates one delay or more than two cautions; and “abnormal” indicates two or more delays [34,35]. In our study, as defined previously [36], we considered a child to have a neurodevelopmental delay if the DDST-II result was either questionable or abnormal. Forty infants were divided into two groups according to the presence or absence of developmental delay; anterior fontanelle size was compared between the two groups.

This study was conducted with approval from the Institutional Review Board and Ethics Committee of Daegu Catholic University Hospital, in compliance with the principles outlined in the Declaration of Helsinki (IRB no: CR-23-160).

Statistical analyses were conducted using IBM SPSS for Windows (version 22.0; IBM Corp., Armonk, NY, USA). The normality of the distribution was assessed using the Shapiro–Wilk test. Intergroup statistical differences were determined using the Kruskal–Wallis test and Mann–Whitney U tests. In cases where the Kruskal–Wallis test indicated a significant difference between groups, a post hoc Mann–Whitney U test with Bonferroni correction was applied. An interclass correlation coefficient was used to examine the intra-rater reliability of repeated anterior fontanelle size measurements. Spearman correlation tests were performed to assess the correlations between age and anterior fontanelle size, as well as between CVA and anterior fontanelle size. Mean values were reported with 95% confidence intervals (CIs). All data were presented as means ± standard deviations. Statistical significance was set at *p* < 0.05. Additionally, a post hoc power analysis was conducted; the power was calculated to be >0.95.

## 3. Results

The infants were divided into groups based on corrected age; the mean age and anterior fontanelle size were determined using ultrasonography and skull AP radiographs for each group (Table 1 and Table 2). On the skull AP radiographs, anterior fontanelle size could not be measured in 76 of the 189 infants (approximately 40%) either because the anterior fontanelle was not clearly visible or because the infants had moved (Table 2). This issue was particularly prevalent among the 0–1-month age group, where measurements were not possible for any patient. A reliability analysis was conducted on 113 infants whose anterior fontanelle sizes were successfully measured using both ultrasonography and AP skull radiographs. The results revealed a significant and reliable correlation between the two measurement methods, with an interclass correlation coefficient of 0.815 (*p* < 0.05).

**Figure 3:**
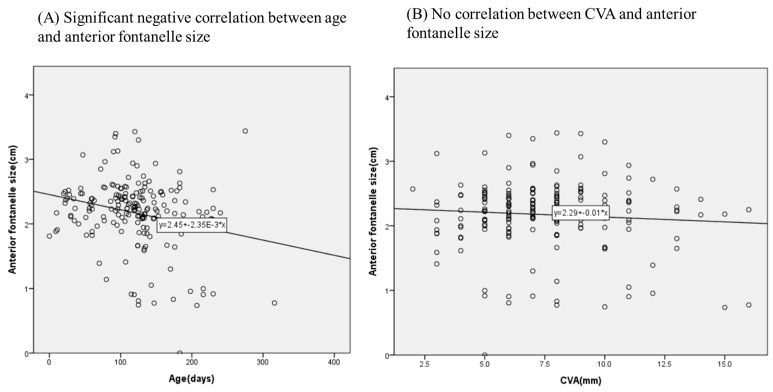
Correlation of anterior fontanelle size with CVA and age. (**A**) A significant negative correlation was observed between age and anterior fontanelle size, with a correlation coefficient of −0.225, as determined by Spearman correlation analysis (*p* < 0.05); (**B**) there was no significant correlation found between CVA and anterior fontanelle size (*p* = 0.989).

A significant negative correlation was observed between age and anterior fontanelle size, with a correlation coefficient of −0.225 (*p* < 0.05). However, no significant correlation was found between CVA and anterior fontanelle size (*p* = 0.989) (Figure 3). The interclass correlation coefficient for repeated measurements of anterior fontanelle size using ultrasonography was 0.923.

Among the three groups categorized according to CVA, there were no significant differences in demographic data, including age, sex, affected side, and risk factors. Additionally, there were no significant differences in anterior fontanelle size between Group 1 (2.11 ± 0.55 cm), Group 2 (2.25 ± 0.48 cm), and Group 3 (2.04 ± 0.62 cm) (*p* = 0.551) (Table 3). The mean grades of DP, CVA, and CVAI for Group 2 (2.48 ± 0.67 grade, 7.34 ± 1.08 mm, 5.28 ± 0.74%) were higher than those of Group 1 (2.00 ± 0.44 grade, 4.28 ± 0.88 mm, 3.13 ± 0.61%) but lower than those of Group 3 (2.78 ± 0.57 grade, 11.61 ± 1.77 mm, 8.18 ± 1.23%) (*p* < 0.05, Table 3).

Of the 40 infants who underwent the DDST-II, 21 were assigned to the non-developmental delay group and 19 to the developmental delay group. There were no significant differences in mean DP, CVA, or CVAI grades between the two groups (Table 4). In the non-developmental delay group, mean anterior fontanelle size was 2.05 ± 0.44 cm, whereas it was 1.48 ± 0.51 cm in the developmental delay group. Nevertheless, there was no significant difference in anterior fontanelle size between the two groups (*p* = 0.09) (Table 4).

## 4. Discussion

This retrospective study aimed to elucidate the relationship between the severity of DP, anterior fontanelle size, and developmental delays in infants. Our findings did not reveal significant differences in anterior fontanelle size among infants stratified according to the severity of DP. This suggests that anterior fontanelle size may not be directly influenced by the severity of DP. However, it is important to note that our study focused solely on anterior fontanelle size and did not assess other potential cranial abnormalities or developmental factors that might contribute to DP severity.

Kim et al. [26] demonstrated that larger anterior fontanelle sizes were associated with greater effectiveness of helmet therapy, owing to the increased movement of the cranial bones. However, their study measured anterior fontanelle size using AP radiographs of the skull, which may be less precise because of insufficient mineralization of the neonatal cranium [37] and due to infant exposure to harmful radiation. In our study, approximately 40% of anterior fontanelle size measurements on skull AP radiographs failed; no measurements could be made in infants aged <1 month. Using ultrasonography, we measured anterior fontanelle size in all infants, regardless of age or movement during the examination. Our study also revealed a high correlation between the two measurements, thus suggesting that ultrasonography is a more accurate and reliable tool for measuring anterior fontanelle size in infants. Additionally, as compared with ultrasonography, the reference point when measuring anterior fontanelle size in skull AP radiographs was ambiguous and subjective, and thus, potentially error-prone. Therefore, using ultrasonography instead of plain radiography can provide more accurate measurements with a lower radiation risk.

In another study, Wendling-Keim et al. [38] observed that patients with a larger anterior fontanelle exhibited a faster rate of cranial remodeling without helmet therapy than those with a smaller fontanelle. This suggests that infants with a smaller anterior fontanelle may have reduced potential for natural cranial correction, making helmet therapy a particularly beneficial intervention for these cases. Therefore, measuring the size of the anterior fontanelle could be crucial for managing infants with DP, as it may provide valuable prognostic information to guide the selection of the most appropriate treatment. The use of ultrasonography for measuring anterior fontanelle size offers a novel approach that could be pivotal for infants with DP. Future studies should aim to validate the effectiveness of this method by comparing the outcomes of spontaneous remodeling and helmet therapy, as reported in other studies.

In our study, we measured the average fontanelle size in each age group of Korean infants with DP, as confirmed through skull radiographs or ultrasonography. This provides detailed, accurate, and age-specific data on anterior fontanelle size in Korean infants, which can serve as a valuable reference for clinicians when assessing normal cranial development.

The observed negative correlation between age and anterior fontanelle size is consistent with previous research, which indicated that anterior fontanelle size gradually decreased with infant age [39,40]. These earlier studies demonstrated that the anterior fontanelle is largest at 1–3 months and continues to decrease thereafter, typically beginning to close at approximately 6–7 months, with significant closure (up to 77%) occurring by 12 months. Although our findings showed that the largest anterior fontanelle size at 3–4 months was slightly different from that previously reported, the general pattern of decrease, following an initial increase, remained consistent. We used ultrasonography to measure anterior fontanelle size, defining it as the longest transverse distance between the skull bones at the frontal head. This methodology differs from those of previous studies, which often relied on anthropometric measurements based on the area or average of the longest vertical and horizontal lines. Additionally, our inclusion of infants with DP may have contributed to discrepancies in the results. Despite these differences, the overall trends in our data were consistent with those observed in previous studies.

Given this context, future studies might benefit from a more comprehensive approach by measuring both the horizontal and vertical distances of the anterior fontanelle using ultrasonography. This would enable the calculation of the average of these two lengths or of the total area of the anterior fontanelle, thus potentially providing a more detailed understanding of its developmental trajectory in infants, particularly in those with DP. Such detailed measurements could refine our understanding of the anatomical changes and help tailor interventions more effectively.

In this study, we found no significant correlation between CVA and anterior fontanelle size. This suggests that although DP may result in cranial asymmetry, it does not necessarily affect the size of the anterior fontanelle. The anterior fontanelle is described as a curved, rhomboid-shaped, non-mineralized, fibrous membrane located at the junction where the coronal, sagittal, and metopic sutures converge in the developing fetus and infant [21]. The metopic suture is the earliest to close, typically beginning as early as 3 months, and is usually completed by 9 months of age. In contrast, other sutures generally do not close until adulthood. Craniosynostosis involves the premature fusion of skull sutures and results in restricted growth and potential deformation of the skull [41,42]. However, we excluded cases of synostosis that could influence the anterior fontanelle size. Additionally, a previous study indicated that anterior fontanelle fusion was not strongly associated with craniosynostosis, with a sensitivity of 38% and a specificity of 61% [43].

These findings highlight the multifactorial nature of the etiology of DP and reinforce the importance of comprehensive assessment methods that go beyond simple measurements of cranial morphology. Future research with larger sample sizes may be necessary to elucidate the complex relationships among cranial asymmetry, suture closure, and fontanelle size, thereby enhancing our understanding of DP and its management.

In this study, we observed no significant differences in anterior fontanelle size between infants with and without developmental delays. Anterior fontanelles have been associated with various medical conditions, including delayed brain growth and developmental disorders. The timing of closure varies significantly between patients and studies. Duc et al. [44] found no significant relationship between the closure time and anterior fontanelle size, suggesting that these two factors may not be directly related. Similarly, Popich and Smith [22] reported that an exceptionally small fontanelle should not be a major concern; the variability in closure timing is considerable. Conversely, earlier research suggested that early closure of the anterior fontanelle might lead to motor developmental delays in infants as young as 7–8 months [24]. A subsequent study confirmed that such early closures could affect the personal–social and cognitive development of children at 16 months [25].

Despite these findings, the relationship between anterior fontanelle size and developmental delay remains unclear. To the best of our knowledge, no previous study has specifically addressed this relationship. Our results suggest that the size of the anterior fontanelle, independent of its closure, may not be a reliable indicator for developmental delay in infants with DP. This may be attributed to several factors, including the relatively young age (<1 year) of most patients included in our study, the lack of long-term follow-up data on developmental outcomes, and the absence of fontanelle closure cases. Additionally, various causes of developmental delays that were not considered in our analysis may also play a role. Therefore, our study underscores the importance of considering multiple factors beyond fontanelle size when assessing developmental risks in infants with DP. Nevertheless, our findings contribute to the body of evidence, suggesting that anterior fontanelle size alone is not a significant predictor of developmental delays, which may alleviate concerns regarding fontanelle size in clinical assessments.

Further research is necessary to explore the potential connections between anterior fontanelle size and developmental delays. Future studies should include larger sample sizes and long-term observational data to better understand these dynamics and to potentially revise current diagnostic and therapeutic practices for infants with DP.

In addition to our primary findings, our study also revealed a high ICC (0.923) for repeated measurements of anterior fontanelle size using ultrasonography. This underscores the reliability of ultrasonography in assessing anterior fontanelle size, supporting its utility in clinical settings. This precise measurement of repeatability is crucial for monitoring the progression or resolution of DP over time and assessing the effectiveness of therapeutic interventions.

The robustness of ultrasonography highlighted in our study aligns with the methodologies applied in related research. Kwon et al. [45] demonstrated the application of ultrasonography for measuring muscle size in patients with congenital torticollis, emphasizing its precision and repeatability across different clinical contexts. Similarly, Park et al. [46] utilized shear wave sono-elastography to evaluate muscle stiffness in infants with congenital muscular torticollis, further illustrating the versatility of ultrasound techniques in pediatric assessments. Furthermore, previous studies have extensively used ultrasonography to analyze changes in skull shape and effectiveness of cranial molding helmet therapy, thus providing a foundation for our methodological approach [14,47]. These studies collectively highlight the evolving role of ultrasonography, not only in diagnosing and managing musculoskeletal abnormalities but also in broader pediatric applications, thereby reinforcing the potential for its application in ongoing and future research on developmental conditions.

Our study had several limitations. First, due to its retrospective nature, we had limited access to medical records for participant evaluation. There is a need to confirm the validity of the results of this study through future prospective studies. Second, the sample size of each group, stratified by age or severity of DP, was insufficient for statistical significance. Third, although anterior fontanelle size can be defined using various methods, we only calculated the longest transverse distance measured using ultrasonography. Future studies should consider additional analyses, such as measuring the vertical distance or area of the anterior fontanelle using ultrasonography. Fourth, because we used only the DDST to determine developmental delay without a long-term follow-up, other diagnostic methods with extended observation periods are necessary. Lastly, we did not consider the prognostic value of anterior fontanelle size in this study. Future research should evaluate the effects of treatments, such as spontaneous remodeling or helmet therapy, on DP from a clinical perspective.

## 5. Conclusions

Our study showed the association of anterior fontanelle size in infants with DP by employing skull AP radiographs and ultrasonography for measurements, a method not commonly used in previous studies. We provided accurate assessments of anterior fontanelle size and offered new insights into its relationship with DP and developmental delays in infants. Our findings demonstrate a significant negative correlation between age and anterior fontanelle size. However, we did not observe any correlation between DP severity and anterior fontanelle size or between anterior fontanelle size and developmental delay. Future research is essential to further understand the clinical implications of these findings and to explore additional and potentially more comprehensive methods for measuring the anterior fontanelle using ultrasonography.

## Figures and Tables

**Table 1:** Mean corrected age and anterior fontanelle size of each age group measured using ultrasonography.

Age Group (n)	Mean Age (Days)	Mean Anterior Fontanelle Size (cm, Range)
0–1 month (n = 13)	19.77 ± 9.38	2.23 ± 0.25 (1.81–2.52)
1–2 month (n = 19)	50.37 ± 9.25	2.26 ± 0.29 (1.82–3.07)
2–3 month (n = 16)	79.25 ± 8.61	2.31 ± 0.53 (1.14–3.12)
3–4 month (n = 47)	106.15 ± 9.15	2.34 ± 0.48 (0.9–3.43)
4–5 month (n = 50)	132.94 ± 7.92	2.12 ± 0.52 (0.75–3.3)
5–6 month (n = 19)	165.74 ± 9.13	2.06 ± 0.45 (0.83–2.52)
>6 month (n = 25)	214.92 ± 31.33	1.86 ± 0.79 (0–3.44)

Values are presented as mean ± standard deviation or number.

**Table 2:** Mean = corrected age and anterior fontanelle size of each age group, as determined via anterior–posterior radiograph of the skull.

Age Group (n)	Detection Rate (n, %)	Mean Anterior Fontanelle Size (cm, Range) *
0–1 month (n = 13)	0/13 (0%)	-
1–2 month (n = 19)	6/19 (31.6%)	2.50 ± 0.83 (1.69–3.98)
2–3 month (n = 16)	7/16 (43.8%)	2.83 ± 0.95 (1.51–4.13)
3–4 month (n = 47)	31/47 (66%)	3.02 ± 0.84 (1.00–5.26)
4–5 month (n = 50)	35/50 (70%)	2.53 ± 0.85 (1.01–4.95)
5–6 month (n = 19)	13/19 (68.4%)	2.51 ± 0.99 (0.92–4.54)
>6 month (n = 25)	21/25 (84%)	2.39 ± 1.21 (0–4.63)
Total (n = 189)	113/189 (59.8%)	

Values are presented as mean ± standard deviation or number. * Mean anterior fontanelle size was calculated only when it was possible to measure it on an anteroposterior radiograph of the skull.

**Table 3:** Demographic and clinical characteristics of infants diagnosed with deformational plagiocephaly.

Variable	Group 1 (n = 43) (CVA ≤ 5 mm)	Group 2 (n = 105) (5 mm < CVA < 10 mm)	Group 3 (n = 41) (CVA ≥ 10 mm)	*p*-Value
Age (days)	114.58 ± 54.30	115.28 ± 53.82	136.80 ± 56.36	0.080
Gender (male/female)	26:17	66:39	30:11	0.411
Affected side (right/left)	20:23	65:40	22:19	0.209
Risk factors				
Oligohydramnios	0	4	2	0.380
Breech delivery	4	9	0	0.143
Twin baby	3	10	3	0.842
Scale (grade)	2.00 ± 0.44 ^†♦^	2.48 ± 0.67 ^†‡^	2.78 ± 0.57 ^‡♦^	0.000 *
CVA (mm)	4.28 ± 0.88 ^†♦^	7.34 ± 1.08 ^†‡^	11.61 ± 1.77 ^‡♦^	0.000 *
CVAI (%)	3.13 ± 0.61 ^†♦^	5.28 ± 0.74 ^†‡^	8.18 ± 1.23 ^‡♦^	0.000 *
Anterior fontanelle size (cm)	2.11 ± 0.55	2.25 ± 0.48	2.04 ± 0.62	0.074

Values are presented as mean ± standard deviation or number; Group 1: infants equal to or less than 5 mm in CVA; Group 2: infants greater than 5 mm and less than 10 mm in CVA; Group 3: infants equal to or greater than 10 mm in CVA; CVA: cranial vault asymmetry; CVAI: cranial vault asymmetry index. *p* values represent between-group comparisons. * *p* < 0.05, Kruskal–Wallis test among groups. † *p* < 0.05, post hoc Mann–Whitney U test with Bonferroni correction between Group 1 and Group 2. ‡ *p* < 0.05, post hoc Mann–Whitney U test with Bonferroni correction between Group 2 and Group 3. ♦ *p* < 0.05, post hoc Mann–Whitney U test with Bonferroni correction between Group 1 and Group 3.

**Table 4:** Comparison of the severity of deformational plagiocephaly and anterior fontanelle size based on developmental delay evaluated by Denver Developmental Screening Test II (DDST-II).

Variable	Developmental Delay (−) (n = 21)	Developmental Delay (+) (n = 19)	*p*-Value
Scale (grade)	2.52 ± 0.51	2.32 ± 0.67	0.421
CVA (mm)	7.05 ± 2.18	6.79 ± 2.90	0.486
CVAI (%)	5.02 ± 1.55	4.91 ± 1.94	0.688
Anterior fontanelle size (cm)	2.05 ± 0.44	1.48 ± 0.51	0.09

Values are presented as mean ± standard deviation. CVA, cranial vault asymmetry; CVAI, cranial vault asymmetry index; DDST, Denver Development Screening Test. *p* values represent between-group comparisons. * *p* < 0.05, Mann–Whitney U test between groups.

## Data Availability

All data generated or analyzed during this study are included in this published article.
